# The Role of Belatacept‐Use and Senescence on Infectious and Mortality Complications After Kidney Transplantation

**DOI:** 10.1111/tid.70175

**Published:** 2026-01-26

**Authors:** Hareesh Singam, Abdinoor Abdi, Wyatt Tarter, Ben Langworthy, Adam Bregman, Karam M. Obeid

**Affiliations:** ^1^ Department of Infectious Disease Cleveland Clinic Foundation Cleveland Ohio USA; ^2^ University of Minnesota Medical School Minneapolis Minnesota USA; ^3^ Medtronic Minneapolis Minnesota USA; ^4^ Division of Biostatistics & Health Data Science University of Minnesota Minneapolis Minnesota USA; ^5^ Division of Nephrology University of Wisconsin Madison Wisconsin USA; ^6^ Division of Infectious Diseases and International Medicine University of Minnesota Minneapolis Minnesota USA

**Keywords:** belatacept, BK, CMV, EBV, kidney transplant

## Abstract

**Introduction:**

Studies evaluating the incidence of infections after belatacept as a substitute for calcineurin inhibitors (CNI) or antimetabolite in kidney transplant (KT) yielded conflicting results. We compared infectious outcomes after belatacept‐use to no belatacept‐use in KT recipients.

**Methods:**

We included patients with primary KT between January 1, 2018, and December 31, 2022. We compared outcomes between those who received belatacept and those who did not. Cox proportional hazards models were used with belatacept as a time‐varying covariate and adjusted for gender, age at transplant, donor type, underlying disease, CMV serostatus, and organ transplant type. Anderson‐Gill hazard of the first Cox models was used to examine the recurrence of DNAemia.

**Results:**

Of 401 KT recipients, 25 received belatacept. Belatacept‐use had a higher hazard for EBV and CMV first infection (3.71 [95% CI 1.57, 8.72; *p* = 0.003] and 2.63 [95% CI 1.04, 6.70; *p* = 0.042], respectively), and for allograft failure (14.5 [95% CI 5.15, 41.0; *p* < 0.001]) and acute cellular rejection (5.08 [95% CI 2.56, 11.5; *p* < 0.001]). Each year increase in zage had a higher hazard for first EBV (3.71 [1.01, 1.05]; *p* = 0.005) and CMV infection (1.02 [1.00, 1.04]; *p* = 0.019). D+/R− CMV serostatus had a higher hazard for first CMV infection relative to other serostatuses (4.96 [3.14, 7.85]; *p* < 0.001).

**Conclusion:**

Careful selection of KT for belatacept‐use is recommended in older recipients (≥ 55 years) due to the increased hazard of mortality, CMV, and EBV DNAemia.

## Graphical abstract

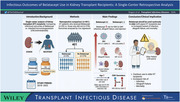

**Abbreviations**
ACR: acute cellular rejection
AMR: antibody‐mediated rejection
BENEFIT study: A Phase III study of belatacept‐based immunosuppression regimens versus cyclosporine in renal transplant recipients.
BKV: BK virus
BKVN: BKV‐associated nephropathy
CMV: cytomegalovirus
CNI: calcineurin inhibitor
cPRA: calculated panel reactive antibody
D+/R−: donor positive, recipient negative
DM: diabetes mellitus
DSA: donor‐specific antibody
EBV: Epstein–Barr virus
IS: immunosuppressive therapy
KS: Kaposi sarcoma
KT: kidney transplant
MMF: mycophenolate mofetil
MPA: mycophenolic acid
OI: opportunistic infections
PJP: *pneumocystis jirovecii* pneumonia
POD: postoperative day
PTLD: post‐transplant lymphoproliferative disease
rATG: rabbit antithymocyte globulin
SD: standard deviation
SOT: solid organ transplant
TMA: thrombotic microangiopathy
UMN: University of Minnesota
UTI: urinary tract infections

## 1 Introduction

Belatacept is a selective co‐stimulation blocker of the effector T‐cell pathway that inhibits T‐cell activation by binding to CD80/86, preventing their interaction with CD28. Belatacept has been used as an alternative to calcineurin inhibitors (CNI) in kidney transplant (KT) recipients, either as a “de novo” (immediately after transplant) or “conversion” (changing from CNI or antimetabolite to belatacept) maintenance immunosuppressive therapy (IS) to prevent CNI‐induced adverse events [1, 2]. Literature provided conflicting evidence on whether belatacept‐use represented a risk for infectious complications. Rates of serious infectious complications were similar in studies comparing the efficacy and safety of “de novo” belatacept IS to the traditional CNI IS [1, 2]. Similar results were observed when evaluating the long‐term outcomes of “de novo” and “conversion” belatacept‐use for maintenance IS [3, 4, 5, 6, 7, 8, 9]. In addition, Neuwirt et al. reported a higher rate of serious infections (defined as infections leading to hospitalization) among patients who received belatacept compared to CNI in univariate analysis only, but not in Cox regression multivariate analysis [10]. These serious infections were diarrheal illnesses, sepsis, and urinary tract infections (UTI), but not viral infections, such as CMV [10]. Non‐serious infections, on the other hand, were reported to be higher with belatacept‐use [8, 9]. A study by Karadkhele showed an association between belatacept IS and CMV infection when compared to CNI IS [11]. Additionally, a study by Chavarot et al. showed an increased incidence of CMV disease in atypical locations such as the anterior and posterior segments of the eye rather than the intestines, particularly in patients older than 70 years, late‐onset disease, and reactivation in CMV seropositive patients converted to belatacept from CNI [12]. In one descriptive study evaluating the rates of opportunistic infections (OIs) after conversion from CNI IS to belatacept IS, 12% of KT recipients experienced OI [13]. Belatacept was also associated with *Pneumocystis jirovecii* pneumonia (PJP) in KT recipients [14].

At the University of Minnesota (UMN), belatacept is used as a conversion option for KT recipients who cannot tolerate CNI or antimetabolite. This study aims to evaluate the rates of infections, including viral, fungal, and parasitic infections, in KT recipients who received belatacept compared to those who did not. We hypothesize that belatacept‐use is associated with higher rates of infectious complications in KT patients.

## 2 Methods

### 2.1 Study Design

This is a retrospective study. Using the solid organ transplant (SOT) database at UMN, we identified adults (≥ 18 years old) with the first episode of KT recipients between January 1, 2018, and December 31, 2022, and allowed for a minimum of 6‐month follow‐up (until June 30, 2023). Only those who received rabbit antithymocyte globulin (rATG) induction therapy were included. Those who received belatacept at any time after transplant, but during the study period, were classified as cases. KT recipients with rATG induction therapy but who have not received belatacept were classified as controls. As there is a black box warning against the use of belatacept in EBV recipient‐negative (EBV R−) serostatus due to the risk of post‐transplant lymphoproliferative disease (PTLD), no EBV R− KT recipients received belatacept. EBV R− patients were also excluded from the control group of the study.

During the study period, induction with rATG for a total dose of 6 mg/kg was given for KT. Doses were decreased by 50% for platelet counts of 50 000–74 000/µL and held for platelet counts less than 50 000/µL. Methylprednisolone doses were 500 mg intraoperatively, 250 mg on postoperative Day 1 (POD1), and 100 mg on POD2. Patients given rATG induction received rapid steroid withdrawal (defined as withdrawal of steroids within 28 days of transplant). Starting March 2020, due to the COVID‐19 pandemic, the induction strategy for KT was changed to be based on the calculated panel reactive antibody (cPRA). Patients with cPRA < 20% with no pre‐existing donor‐specific antibody (DSA) were given basiliximab 20 mg intraoperatively and on POD4 with methylprednisolone 500 mg intraoperatively, 250 mg on POD1, 100 mg on POD2, followed by a rapid steroid withdrawal. Patients with cPRA 20%–80% were given rATG 2 mg/kg intraoperatively, basiliximab 20 mg on POD1 and POD5, methylprednisolone 500 mg intraoperatively, 250 mg on POD1, 100 mg on POD2, followed by rapid steroid withdrawal. Patients with cPRA > 80% (or delayed graft function or DSA) received rATG 6 mg/kg with early steroid withdrawal (defined as within 5 days of transplant) as prior. All patients were started on tacrolimus on POD 0 with a trough goal of 8–10 ng/mL for 6 months, 6–8 ng/mL until 1‐year post‐transplant, and 4–6 ng/mL thereafter. Mycophenolate mofetil (MMF) 1000 mg IV was given intraoperatively to all patients, followed by 750 mg q12h to maintain trough levels between 1 and 3.5 mcg/mL. Indications to transition from CNI or MMF/mycophenolic acid (MPA) to belatacept (conversion) were thrombotic microangiopathy (TMA), poor graft function with biopsies negative for rejection, severe arteriolar hyalinosis on biopsy with poor graft function, and the inability to tolerate side effects of CNI or MMF/MPA. The decision to transition to belatacept was at the discretion of the treating physician.

In addition to the information provided by the SOT database, the authors (A.A. and K.M.O.) also performed a chart review when they felt it was indicated. For additional inclusion/exclusion criteria, please review the Supporting Information. We collected demographic data, whether the main underlying disease that led to transplantation was diabetes mellitus (DM) versus any other reasons, high‐risk CMV serostatus (D+/R−) versus other, viral infections that included CMV, EBV, and BKV, fungal infections, and rejections. We also sought to look for other OI including toxoplasmosis and Kaposi sarcoma (KS). For CMV, EBV, and BKV infections, we collected data pertaining to the first incidence of DNAemia, recurring viremic events. We also evaluated infection‐related complications such as PTLD and rituximab‐use for EBV infections, and BKV‐associated nephropathy (BKVAN) for BKV infections. Please refer to the Supporting Information for additional definitions. We also evaluated non‐infectious outcomes including mortality, acute cellular rejection (ACR), antibody‐mediated rejection (AMR), and allograft failure.

CMV prophylaxis entailed valganciclovir 900 mg daily (adjusted to creatinine clearance) at the time of transplant and continued for 6 months for high‐risk serostatus (CMV D+/R−) and for 3 months for all other CMV serostatus groups, including CMV D−/R−. In addition, high‐risk CMV serostatus patients are also monitored monthly for CMV viremia using COBAS AmpliPrep/COBAS TaqMan CMV PCR. In addition, all patients were checked for CMV DNAemia if they were symptomatic or with White Blood Cell < 2 × 10^3^/µL. At UMN, EBV R+ KT recipients were monitored for EBV DNAemia at the discretion of the treating physician. All patients are monitored for BKV DNAemia monthly using real‐time TaqMan PCR for the first year after transplant, then every 3 months for another year. Using belatacept was not associated with any change to the above‐mentioned monitoring interventions.

CMV viremia is treated with either valganciclovir or ganciclovir at the recommended induction dose (adjusted to kidney creatinine clearance) until viral load is < 137 IU/mL, followed by maintenance dose as recommended for a total duration of 2 months of therapy. MMF/MPA is typically reduced by half for the first 1–2 weeks. When antiviral therapy is discontinued, CMV viral load is monitored weekly for 1 month, then every other week for 2–3 months. Variation of this approach is at the discretion of the treating physician. When EBV or BKV viremia is detected, IS therapy is reduced at the discretion of the treating physician. In the case of EBV viremia, screening for lymphoma if the patient is symptomatic or if VL is > 5 000 000 IU/mL or if VL is exponentially rising by ≥ 0.5 log/month. The use of rituximab is done in conjunction with an infectious disease consultation. EBV viremia is then monitored monthly for 1 year, then quarterly for another year, then yearly. BKVAN is usually treated in consultation with an infectious diseases specialist with multiple modalities, including decreasing IS, intravenous immunoglobulin, and cidofovir.

Screening for rejection at UMN is at the discretion of the managing physician.

Please see the Supporting Information for the definitions of DNAemia episodes and recurrences.

### 2.2 Statistical Analyses

To examine the association between receiving belatacept after transplant and the incidence of outcomes of interest (mortality, fungal infections, organ rejection, ACR, AMR, and CMV, EBV, and BK viremia infections), Cox proportional hazards models were fit, treating belatacept as a time varying covariate to account for changes in hazard after beginning belatacept (or to account for any initial infections patients received prior to starting belatacept). Models were adjusted for gender, age at transplant, donor type (alive vs. deceased), primary disease category (DM vs. other), and CMV serostatus. To examine the association between receiving belatacept and recurrence of the DNAemia outcomes, Anderson‐Gill Cox models were fit, again treating belatacept as a time varying covariate to incorporate information on the hazards of infection post belatacept. We used age as a continuous variable in our statistical analysis. All analyses were conducted in R version 4.3.1, and alpha for significance was set a priori at 0.05.

## 3 Results

During the study period, we identified 401 unique first KT recipients. Twenty‐five of these 401 (6.2%) received belatacept for “conversion” IS and were considered cases. No one received “de novo” belatacept IS. The rest (376) were classified as controls.

Table 1 summarizes patient demographics, transplant‐, and belatacept‐related information. In summary, in the belatacept group, the mean [±standard deviation (SD)] duration between transplant and the first dose of belatacept was 277 (± 366) days, with the majority of patients [15/25 (60%)] receiving early belatacept (within 6 months of transplant). The mean duration of belatacept was 16 (± 18) months for a mean total dose of 19 (± 19) doses, Table 1.

**TABLE 1:** Characteristics of patients included in the study.

Characteristics	Overall *N* = 401	Control *N* = 376	Belatacept *N* = 25
Age^a^ (years)			
Mean (± SD)	54 (± 14)	53 (± 15)	61 (± 11)
Median (IQR)	56 (43–66)	55 (43–65)	61 (55–70)
Gender			
Male	229 (57%)	214 (57%)	15 (60%)
Female	172 (43%)	162 (43%)	10 (40%)
Donor type			
Alive	239 (60%)	223 (59%)	16 (64%)
Deceased	162 (40%)	153 (41%)	9 (36%)
Primary disease leading to transplant			
DM	81 (20%)	75 (20%)	6 (24%)
Other	320 (80%)	301 (80%)	19 (76%)
CMV serostatus			
D+/R−	80 (20%)	79 (21%)	1 (4%)
Other	321 (80%)	297 (79%)	24 (96%)
Time to first dose belatacept from transplant (days)			
Mean (± SD)			277 (± 366)
Median (IQR)			96 (62–331)
Belatacept timing in relation to transplant			
Early^b^			15 (60%)
Late^c^			10 (40%)
De novo versus conversion therapy			
De novo			0 (0%)
Conversion			25 (100%)
Total duration of belatacept‐use (months)			
Mean (± SD)			16 (± 18)
Median (IQR)			7 (3–27)
Total belatacept doses			
Mean (± SD)			19 (± 19)
Median (IQR)			11 (5–30)

Abbreviations: DM, diabetes mellitus; IQR, interquartile; SD, standard deviation.

^a^ Age at the time of transplant.

^b^ First dose of belatacept ≤ 6 months after transplant.

^c^ First dose of belatacept > 6 months of transplant.

### 3.1 CMV Infection

Incidence of CMV DNAemia: receiving belatacept (aHR 2.63 [1.04, 6.70; *p *= 0.042]), yearly increase in age (aHR 1.02 [1.00, 1.04], *p *= 0.019) and high‐risk CMV serostatus (aHR 4.96 [3.14, 7.85; *p* < 0.001]) were associated with an increased hazard of at least one episode of CMV DNAemia (Table 2). The hazard for CMV DNAemia was lower in those who received an organ from a deceased donor (aHR 0.59 [0.36, 0.96; *p* = 0.032]), Table 2.

**TABLE 2:** Adjusted hazard ratio (aHR) for infectious outcomes of interest and mortality.

		CMV^a^		EBV^a^		BKV^a^		Fungal^a^		Mortality	
		aHR (95% CI)	*p*	aHR (95% CI)	*p*	aHR (95% CI)	*p*	aHR (95% CI)	*p*	aHR (95% CI)	*p*
Control		**—**		**—**		**—**		**—**		**—**	
Belatacept‐use		2.63 (1.04, 6.70)	**0.04**	3.71 (1.57, 8.72)	**< 0.003**	1.02 (0.32, 3.25)	> 0.90	2.86 (0.62, 13.10)	0.20	4.72 (1.85, 12.00)	**< 0.001**
Age^b^, ^c^		1.02 (1.00, 1.04)	**0.02**	1.03 (1.01, 1.05)	**0.005**	1.02 (1.01, 1.04)	**0.004**	1.07 (1.13, 1.10)	**0.006**	1.05 (1.02, 1.13)	**0.003**
Age ≥ 55		1.53 (0.96, 2.46)	0.08	2.12 (1.28, 3.51)	**0.003**	1.30 (0.86, 3.51)	0.20	6.36 (1.44, 28.10)	**0.02**	5.10 (1.77, 14.7)	**0.003**
Gender	Male Female	**—**		**—**		**—**		**—**		**—**	
		0.80 (0.49, 1.29)	0.40	1.01 (0.63, 1.66)	> 0.90	0.80 (0.53, 1.21)	0.30	0.79 (0.30, 2.08)	0.60	0.96 (0.46, 1.99)	> 0.90
Donor type	Alive Deceased	**—**		**—**		**—**		**—**		**—**	
		0.59 (0.36, 0.96)	**0.03**	0.89 (0.55, 1.43)	0.60	0.50 (0.32, 0.79)	**0.003**	0.53 (0.19, 1.49)	0.20	0.57 (0.26, 1.24)	0.20
Primary disease leading to transplant	DM	**—**		**—**		**—**		**—**		**—**	
	Other	1.77 (0.93, 3.36)	0.08	1.24 (0.68, 2.26)	0.50	1.45 (0.84, 2.49)	0.20	0.64 (0.24, 1.70)	0.40	0.53 (0.25, 1.10)	0.09
CMV serostatus	Other D+/R−	**—**		**—**		**—**		**—**		**—**	
		4.96 (3.14, 7.85)	**< 0.001**	1.50 (0.86, 2.60)	0.20	1.18 (0.72, 1.92)	0.50	0.93 (0.27, 3.27)	> 0.90	1.37 (0.58, 3.24)	0.50

^a^ The first incidence of CMV, EBV, BKV, and fungal infections.

^b^ Age at the time of transplant.

^c^ aHR was assessed based on the yearly increase in age.

Recurrence of CMV DNAemia episodes: yearly increase in age (aHR 1.02 [1.00, 1.03], *p* = 0.018) and high‐risk CMV serostatus (HR 7.73 [5.28, 11.3; *p* < 0.001]) are associated with increased hazard of recurring CMV DNAemia, Table 3. Hazard of recurring CMV DNAemia was lower among those who received organs from deceased donors (aHR 0.58 [0.39, 0.87; *p* = 0.008]), Table 3. There was not a statistically significant increased hazard of recurring CMV viremic episodes with belatacept‐use (aHR 1.91 [0.87, 4.19; *p* = 0.11]).

**TABLE 3:** Adjusted hazard ratio (aHR) for recurring DNAemia episodes.

		CMV		EBV		BKV	
		aHR (95% CI)	*p*	aHR (95% CI)	*p*	aHR (95% CI)	*p*
Control		**—**		**—**		**—**	
Belatacept‐use		1.91 (0.87, 4.19)	0.11	2.50 (1.19, 5.29)	**0.02**	0.49 (0.16, 1.56)	0.20
Age^a^, ^b^		1.02 (1.00, 1.03)	**0.02**	1.01 (1.00, 1.03)	0.11	1.03 (1.01, 1.04)	**< 0.001**
Age ≥ 55		1.30 (0.90,1.90)	0.20	1.76 (1.0,3.08)	**0.05**	1.44 (0.97,2.15)	0.07
Gender	Male Female	**—**		**—**		**—**	
		0.86 (0.58, 1.25)	0.40	1.07 (0.68, 1.67)	0.80	1.03 (0.70, 1.52)	0.90
Donor type	Alive Deceased	**—**		**—**		**—**	
		0.58 (0.39, 0.87)	**0.01**	0.95 (0.61, 1.48)	0.80	0.67 (0.44, 1.00)	0.05
Primary disease leading to transplant	DM	**—**		**—**		**—**	
	Other	1.12 (0.71, 1.577)	0.60	1.32 (0.73, 2.04)	0.40	1.22 (0.75, 1.98)	0.40
CMV serostatus	Other D+/R−	**—**		**—**		**—**	
		7.73 (5.28, 11.3)	**< 0.001**	1.44 (0.86, 2.41)	0.20	1.33 (0.85, 2.08)	0.20

^a^ Age at the time of transplant.

^b^ aHR was assessed based on the yearly increase in age.

### 3.2 EBV Infection

Incidence of EBV DNAemia in those receiving belatacept (aHR 3.71 [1.57, 8.72; *p* = 0.003]) and yearly increase in age (aHR 1.03 [1.01, 1.05] *p* = 0.005) were associated with an increased hazard of EBV DNAemia, Table 2.

With regards to recurrence of EBV viremic episodes, belatacept‐use was associated with increased hazard of recurrent EBV viremic episodes (aHR 2.50 [1.19, 5.29; *p* = 0.016]), Table 3.

There was no PTLD in the belatacept or control groups. None of the recipients during the study period received rituximab.

### 3.3 BKV Infection

Incidence of BKV DNAemia: one year increase in age (aHR 1.03 [1.01, 1.04], *p* = 0.004) but not belatacept‐use (aHR 1.02 [0.32, 3.25; *p* > 0.9]) is associated with higher hazard for at least one BKV DNAemia. However, a deceased donor (aHR 0.50 [0.32, 0.79; *p* = 0.003]) is associated with lower hazard for BKV Viremia, Table 2.

Recurrence of BKV DNAemia: there was no significant association between belatacept‐use and recurrence of BKV DNAemia (aHR 0.49 [0.16, 1.56; *p* = 0.2]), Table 3.

BKVAN occurred in a total of four patients, all four patients were in the control group (4/528; 0.76%).

### 3.4 Fungal Infections

After adjusting, the hazard of fungal infection was higher for each one‐year increase (≥ 55 years old) in age (aHR 1.07 [1.13, 1.10], *p* = 0.006).

Description of fungal infections: a total of 18/401 recipients developed fungal infections. 3/25 (12%) in the belatacept group (2 invasive candidiasis [1, abdominal abscess and one candida esophagitis], 1 cryptococcosis) and 15/401 (3.7%) in the controls (4 invasive candidiasis [2 candidemia, 1 abdominal infection, 1 esophagitis], 4 aspergillosis, 3 blastomycosis, 2 histoplasmosis, 1 cryptococcosis, and 1 *Fusarium*).

### 3.5 Other

During this study, there was a single *Mycobacterium tuberculosis* infection in the control group. We did not detect toxoplasmosis parasitic infection, neither did we detect KS.

### 3.6 Rejection and Allograft Failure

Belatacept‐use has a higher hazard for ACR compared to controls (aHR 5.43 [95% CI 2.56, 11.5; *p* < 0.001]). No antibody‐mediated rejection occurred in the belatacept group, so this could not be assessed. After adjusting, the hazard of graft failure was 18.9 (95% CI 6.47, 55.1; *p *< 0.001) times higher for belatacept patients than control patients. Yearly increase in age was associated with lower risk of graft failure [aHR 0.97 (0.94, 1.00), *p* = 0.032].

#### 3.6.1 ortality

After adjusting, the hazard of death was 4.72 (95% CI 1.85, 12.0; *p* = 0.001) times higher for belatacept patients than controls and was 1.05 (95% CI 1.02, 1.13; *p* = 0.003) times higher for each 1‐year increase in age.

## 4 Discussion

This retrospective study evaluated CMV, EBV, BKV, and fungal infections in KT who received belatacept “conversion” IS compared to those who did not. Belatacept and yearly increase of age were associated with an increased hazard for the first incidence of CMV and EBV DNAemia as well as mortality. A yearly increase in age was also associated with a higher hazard for recurrent CMV and BKV DNAemia as well as fungal infections. Belatacept‐use on the other hand was associated with higher hazard for recurring EBV but not CMV and BKV DNAemia.

Belatacept is a fusion protein that inhibits T‐lymphocyte activation by blocking costimulation. Since T cells are crucial for viral control, their inhibition leads to prolonged viral infections, as T‐cell responses are unable to clear the virus [15]. This mechanism likely accounts for the EBV and CMV infectious complications observed with the use of belatacept in the medical literature. The risk of belatacept‐use leading to EBV infection is well‐established, leading to the FDA to restrict belatacept to EBV‐positive recipients. Our study showed higher rates of both CMV and EBV DNAemia and recurrent EBV viremic episodes with belatacept‐use, but no higher hazard for recurrent CMV DNAemia, further corroborating the significant linkage between belatacept‐use and EBV infection. Bassil et al. have also shown a stronger relation between belatacept‐use and EBV compared to CMV DNAemia with a follow‐up period of up to 36 months [16]. In our study, these EBV‐related events did not result in PTLD, an attestation to the successful strategy of avoiding belatacept‐use in recipients with EBV‐negative serostatus. It is worthwhile to mention that the sample size (only 25 KT receiving belatacept) may have resulted in the lack of association between belatacept‐use and recurring CMV DNAemia based on the *p* value, even though the aHR was high at 1.9 (95% CI 0.87, 4.19).

Unlike EBV DNAemia, which, apart from PTLD, is not usually consequential for EBV‐seropositive SOT recipients, CMV infection requires medical attention and an effective prophylactic strategy with antivirals to prevent CMV disease. Though belatacept‐use was not associated with CMV infection in earlier studies [1, 2, 3, 16], CMV infection has been recognized as a major complication in other studies [11, 17]. In addition to the traditional risk factors for CMV infection (i.e., high‐risk CMV serostatus), our study showed a higher incidence of CMV DNAemia, but not recurring CMV viremic episodes in the KT recipients who received belatacept for conversion IS. Yearly increase in age was also associated with increased hazards of CMV infection. These results were corroborated in a study by Chavarot et al., where belatacept‐use as a conversion therapy, older age, and high‐risk CMV serostatus were also risk factors for CMV infection [17]. On the other hand, one study by Marvin et al. showed that infections due to bacterial pneumonia and pyelonephritis, but not CMV infection, were associated with the conversion use of belatacept [18]. This might be attributed to matching cases and controls in that study based on transplant date, age, induction therapy, and CMV serostatus, whereas we included all KT apart from those who did not receive rATG for induction.

CMV is a difficult challenge to manage in the post‐transplant period. A viewpoint article by Zuber et al. hypothesizes that belatacept liberates PD‐L1 from PD‐L1/CD80 heterodimers, increasing CD28‐independent PD‐1‐mediated T‐cell suppression, leading to impairment and exhaustion of CMV‐specific T cells, which could be rescued by mTOR inhibitor therapy [19]. They advocate for belatacept/mTOR inhibitor‐based conversion immunosuppression in a staggered manner to avoid late CMV disease. Further research is needed to evaluate the optimal management of CMV DNAemias after belatacept conversion.

Belatacept‐use did not have a significant effect on BKV infectious complications, consistent with other studies. For instance, the BENEFIT study found no statistically significant difference in BKV infections between belatacept and cyclosporine at 12 months; another ad hoc analysis of the BENEFIT study in one participating center showed no statistically significant difference in BKV DNAemia up to 36 months [2, 16].

Our study did not show a higher risk for invasive fungal infections in KT who received belatacept as conversion IS. This contrasts with other findings in the literature. In the setting of belatacept conversion, a prospective study found that the fungal infections 12 months after belatacept were more frequent with belatacept (11 patients [13%]) than with CNI therapy (three patients [3%]). This was also noted at a 3‐year analysis of the belatacept group (9.73 per 100 p‐y) than in the CNI group (2.58 per 100 p‐y). In this study, the most common fungal infections were skin infections and were mild and did not require any change in immunosuppression [4]. It is likely that our findings differ from those of this study due to the different definitions of fungal infections; we included only opportunistic fungal infections, endemic fungal infections, and invasive candidiasis proven by EGD or biopsy, which is drastically different from simple skin infections. The hypothesis for increased fungal infections on belatacept is likely due to betalacept's inhibition of T cells, which play a role in surveillance and identification of fungal infections as well [20]. In our study, opportunistic fungal infections were numerically higher in the belatacept group (3/25, 12%) than 21/528 (4.2%) in the control group.

Our findings also highlight the importance of advanced age and its association with major complications after KT. We found that a 1‐year increase in age was associated with higher rates of fungal infections, CMV, EBV, and BKV DNAemia, and recurring CMV and BKV viremic episodes. Older adults are often at a higher risk of infections due to immunosenescence, frailty, functional impairment, and multiple comorbidities [21]. In one study, CMV DNAemia was more frequent in older adults (older than 65 years old) in the 1–6 months post‐transplant period [21]. It is important to note that per post‐transplant CMV protocols, this center utilizes pre‐emptive monitoring in the CMV recipient‐seropositive population.

Our data also indicate a higher incidence of ACR with belatacept‐use, consistent with findings from the BENEFIT trial, where belatacept recipients experienced a higher incidence and severity of acute rejection episodes. Most acute rejection episodes in BENEFIT occurred within the first 3 months, did not recur, were not antibody mediated, and generally resolved with treatment [2, 22]. Yearly increase in age was not associated with higher ACR, which could be due to immunosenescence that has the opposite effect on rejection when compared to infections. This immunosenescence may also explain the protective effect of older age on the graft function. Belatacept‐use was an independent risk for graft failure.

Belatacept‐use and yearly increase in age were associated with increased hazard of mortality in our study. Comparing this to the final analysis of the BENEFIT trial data, seven years after transplant, patient and graft survival were significantly higher in the BENEFIT trial with than with cyclosporine. This could be due to the older age of the participants in our study (meaning age of 60 years) compared to the participants in the BENEFIT trial (approximately 43 years).

It is important to acknowledge the limitations of this study, including its retrospective design, limited sample size, and reliance on data from a single center, which is likely subject to human errors entering and interpreting data. Other potential limitations include belatacept‐use for conversion IS, which made belatacept‐use a time‐dependent covariant. Also, the two groups included in the study may have not been similar since it is possible that the belatacept group had CNI‐ or MMF‐related adverse events that may have made them prone to infectious and non‐infectious complications including mortality. In addition, we also did not evaluate the timing of infections in relation to rejection. Another limitation is that patients converted to belatacept may have been more closely monitored for infections, including CMV, EBV, BKV, and fungal infections; however, our belatacept conversion protocol does not indicate the need for more monitoring, which may have been done only based on the treating physician's discretion. Additionally, CNI and/or MMF/MPA trough values were not available in this retrospective study. We made our best effort to mitigate any modifiable limitations by performing an extensive chart review to confirm the SOT database findings. We included only KT recipients who received rATG to minimize the differences between groups. We also fitted Cox proportional hazards models, Adnerson‐Gill Cox models, and the linear mixed‐effect model to treat belatacept as a time‐varying covariate to account for events of interest that may have occurred prior to starting belatacept.

Our study is important and unique as it is one of the very few studies evaluating the infectious complications after belatacept‐use for conversion IS after KT, including recurring viremic episodes and addressing other non‐infectious outcomes such as ACR, graft survival, and mortality.

Clinicians should carefully weigh the potential benefits and risks of belatacept conversion IS, particularly in older SOT recipients. We wonder whether using belatacept may have substituted certain CNI‐ and/or MMF‐related adverse events for others. Transplant centers that use belatacept for conversion IS should consider CMV monitoring, particularly in CMV discordant and older patients. Future research with larger, multicenter cohorts and longer‐term follow‐up may provide additional insights into the infectious risks associated with belatacept conversion IS in SOT recipients. A comparative study looking at the use of belatacept as a conversion versus de novo therapy would be helpful in assessing whether our findings of infectious complications are attributable to belatacept.

## Funding

Statistical analysis was funded and supported by theNational Center for Advancing Translational Sciences of the National Institutes of Health, Award Number UM1TR004405. The content is solely the responsibility of the authors and does not necessarily represent the official views of the National Institutes of Health. No other aspect of the study was funded.

## Ethics Statement

The University of Minnesota Institutional Review Board approved this study.

## Supporting information




**Supporting Figure 1**:tid70175‐sup‐0001‐SuppMat.docx
