# Supplementary material for: The Role of Belatacept‐Use and Senescence on Infectious and Mortality Complications After Kidney Transplantation
Source: Transpl Infect Dis. 2026 Jan 26;28(2):e70175. doi: 10.1111/tid.70175 (PMC13070013; doi:10.1111/tid.70175)
Supplement: Supplementary file 1 — Supporting Figure 1:tid70175‐sup‐0001‐SuppMat.docx [file TID-28-e70175-s001.docx]

**Supplement**

Inclusion criteria:

- All adult (≥18) SOT recipients whose first kidney transplant between 1/1/2018 and 12/31/2022.

Exclusion criteria:

- Any patient younger than 18 years old at the time of transplantation.

- If the patient opted out of research.

- Any patient with any transplant before 1/1/2018.

- Any prior SOT.

- If with a second SOT during the study period.

- Any kidney transplant recipients who did not receive ATG for induction therapy within the study period.

- Any KT recipients who are EBV R-

Definitions

1. Opportunistic infections: listed in the SOT database. Authors may have to review charts to confirm.
   1. Here we are collecting the first diagnosis of each individual opportunistic infection (histoplasmosis, blastomycosis, etc…) not only the first opportunistic infection in general.
2. CMV DNAemia episode is when CMV viral load is between ≥ 137 IU/mL and <137 IU/mL or undetected. The number of recurrences is the total number of CMV DNAemia episodes following the first episode.  Authors reviewed charts.
3. EBV DNAemia episode is when EBV viral load is between ≥500 copies/mL and <500 copies/mL or undetected. The number of recurrences is the total number of EBV DNAemia episodes following the first episode. Authors reviewed charts.
4. BKV DNAemia episode is when BKV viral laod is between ≥500 copies/mL and <500 copies/mL or undetected. The number of recurrences is the total number of BKV DNAemia episodes following the first episode. Authors reviewed charts.
5. Death during study period including follow up period.
6. ACR and AMR in kidney screened for at the discretion of the treating physician. We included those proven by biopsy results, BANFF criteria 2003.
7. Graft dysfunction: rise in creatinine.
8. Graft failure: need for retransplantation evaluation or dialysis.
9. De novo belatacept: belatacept within 2 days of transplant.
10. Early belatacept: first dose started ≤ 6 months after transplant
11. Late belatacept: >6 months after transplant.
12. Fungal infections only included opportunistic infections (histopasmosis, blastomycosis, cryptococcosis, aspergillosis, *Pneumocystis jirovecii* pneumonia, other opportunistic fungal infections) in addition to invasive candidiasis fulfilling the EORTC criteria in addition to candida esophagitis proven by EGD. This excludes candiduria and oral thrush due to inability to confirm its clinical significance based on chart review, but included candida esophagitis proven by EGD with or without biopsy. Also excluded skin and soft tissue infections such as onychomycosis and tinea corporis.
